# A dual switched DC-DC converter with high gain for low-voltage fuel cell stack electric vehicles

**DOI:** 10.1038/s41598-025-29581-3

**Published:** 2025-11-27

**Authors:** Athikamsetti Rudra Santhosh Kumar, Arun Nandagopal

**Affiliations:** School of Electrical Engineering, Vellore Institute of Technology, Vellore, Tamil Nadu 632014 India

**Keywords:** Low voltage fuel cell stack, Capacitor clamped, Current ripple, Switched capacitor (SC), Switched inductor (SI), Voltage gain, Engineering, Electrical and electronic engineering

## Abstract

A high-gain, boost-derived, non-isolated DC-DC converter employing dual switches is introduced to supply the 400 V DC link bus voltage required for fuel cell vehicles, featuring a minimal number of passive components and diodes. Additional features of a DC-DC converter employed in fuel cell electric vehicles include minimal input current ripple, attenuated inrush currents, lowered voltage stress, and minimized reverse recovery losses, all contributing to a reduction in the converter’s size and weight. The proposed converter achieves the aforementioned characteristics that conventional converters cannot replicate. The voltage gain of this converter, varying from 20 to 8, is achieved with a low wide variation of input voltage between 20 V and 50 V. This article addresses the functionality, control methods, and characteristics of comparable converters. The suggested converter is validated through hardware, providing an output power of 400 W/400 V at a 1 A load across a low-wide fluctuation in input voltage (20 V to 50 V) at a switching frequency of 50 kHz.

## Introduction

 In recent years, there has been a rise in the production of cars powered by environmentally friendly energy, and the percentage of the overall transportation market has been expanding^1^.

**Fig. 1:**
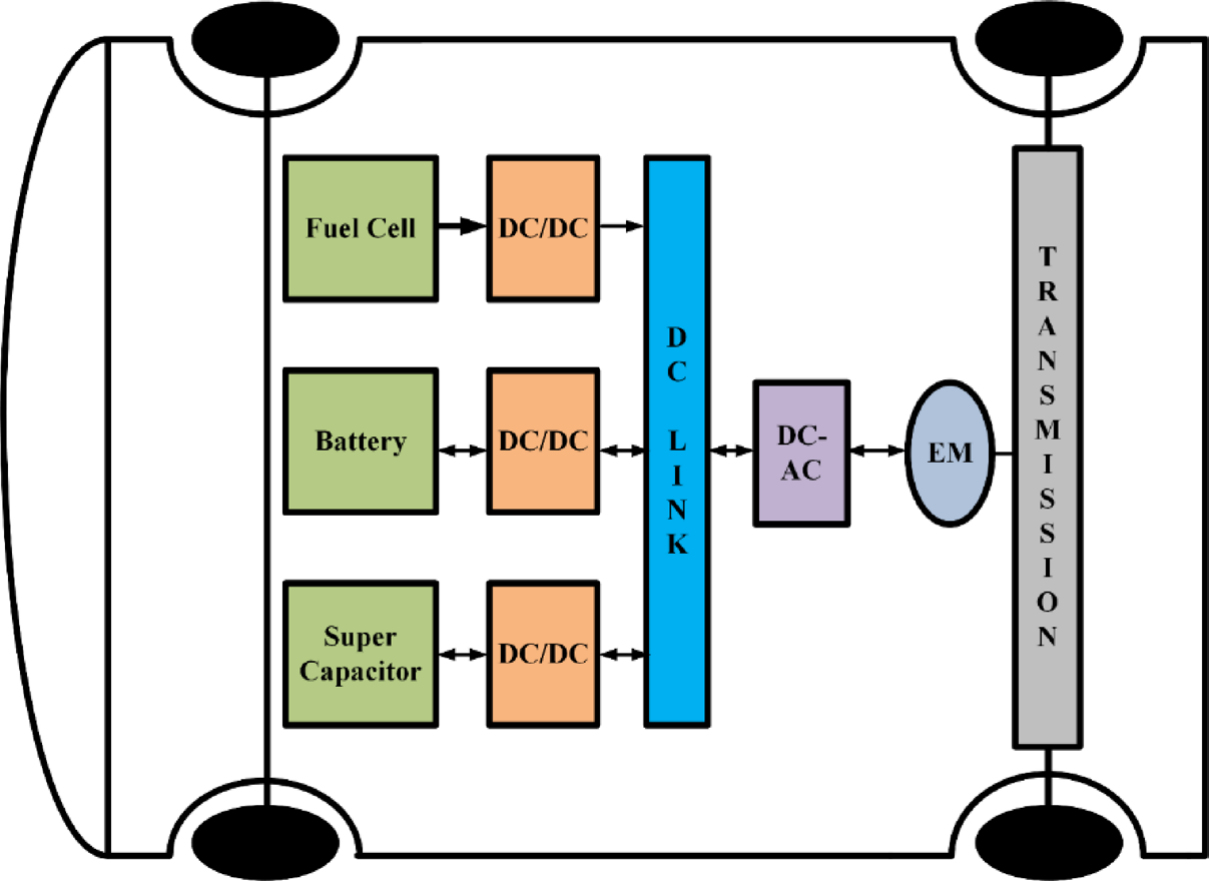
Generalized block diagram of clean energy electric vehicle (EV).

Fuel cell-powered cars are a crucial component of the clean energy-based transportation networks. Since fuel cell-powered vehicles generate clean power, run smoothly, and have a high-density current output, their use is increasing rapidly^2^. However, unlike batteries^3,4^ that have a constant output voltage, the output voltage of the fuel cell drops when there is a sudden increase in current at the output. In order to provide a constant 400 V DC-link bus voltage, it is therefore necessary to interface the fuel cell stack to a high voltage gain dc to dc converter ‎^5^, and the block diagram of the generalized fuel cell-based electric vehicle is shown in Fig. 1.

The different topological structures of DC-DC converters based on the isolation and non-isolation types described in^6^ achieve the output voltage for the variation of input voltage. Though the output voltage is reached, isolated converters require high-frequency transformers to transmit power from input to output. Multiple conversion/inversion operations are required to convert DC-AC-DC due to the transformer. Additionally, because isolated converters use a transformer, they produce additional core losses, EMI effects, voltage spikes, and a rise in topology size^7^. Hence, non-isolated converters are preferred to isolated converters.

At high gain, the typical boost converter exhibits substantial voltage stress. Consequently, other types of non-isolated high gain converters such as cascaded/multilevel, interleaved structure, coupled inductors, switched-inductor (SI)/switched capacitor (SC), and Z-source converters are reported in the introduction.

Cascaded converters^8,9^ and multiport converters^10^ overcome the conventional converter issues. But this type of converter requires more than one converter for the high voltage gain with a complex control circuit and requires more components.

In the coupled inductor converter^11^, though the voltage gain increases with the number of turns of the inductor, the voltage stress on the capacitor and diode increases with a significant reduction of voltage stress across the switch. However, the gain is high; the increase in dimension of the circuit topology increases the converter cost.

The interleaved structure in^12^ is based on parallel-series combination circuit topology at the input and output sides, respectively. The input current ripple is nearly zero, but the control input is to be varied within certain limits due to the interleaving structure. The interleaved structure presented in^13^ operates with the combination of magnetic coupling-multiplier-voltage lift, which gives rise to high gain but with a decrease in efficiency at low power loads. The interleaved converter presented in^14^ with a greater number of phases and operating with a switched inductor achieves high gain, but the component count increases when the number of phases is increased, and consequently the weight of the converter is also increased. The multi-phase converter^15^ is restructured as a modular converter to increase the gain further with capacitor and inductor self-balancing voltages and averaging current techniques, respectively, at the cost of increased weight of the topology. To alleviate the dimension of the multi-phase converter^16^, the coupled inductor is replaced by a permanent magnet. To overcome the above issues, the interleaved structure^17,18^ performance is enhanced through soft switching by using additional auxiliary circuits.

The switched capacitors and switched inductor-type converters^19–21^ were introduced to increase the voltage gain with a lower value of the control input. The ripple in the input current is significant, and voltage fluctuations appear across the controlled switches. In^22^, the ripple current is significantly reduced by using a clamped capacitor at the input, and the utilization of a switched capacitor (SC) at the output side gives high voltage gain at reduced stress across semiconductor devices. The modified structure of SI and SC presented in^23^ is operated at lower control input but uses two active controlled switches with a common ground point between input and output.

The impedance type of power converters^24^ in the form of a ‘y’ shape achieves limited voltage gain and uses one inductor in each leg of the ‘y’ network besides the input inductor. Hence, the topology is of more than considerable size.

An enhanced gain with the combination of boost capability and a quasi-z-source network is reported in^25^. Even though the total component of the topology is 11, only one active switch is utilized. Moreover, with the utilization of three inductors in the topology, the weight of the power circuit increases proportionately.

The triple network in the form of a ‘z’ power converter^26^ reaches higher gain by operating in both continuous mode and discontinuous mode. Among the triple networks, the first and last part act as boost modes, and the middle part is the switching network. Due to two boosting modes, the passive components are more, which contributes more weight.

The power converter^27^, derived by integrating two quasi-z-source interleaved structures at the input side and a passive element at the load side, realizes high gain such that the control input is less than 0.5. The merit of this topology is that high gain is achievable at the cost of wide variation of the output of the fuel cell, which is interfaced to the topology. Similarly, hybrid topology with ‘z’ network is shown in^28^.

A power converter utilizing a single controlled switch with multiple passive components and uncontrolled switching devices portrayed in^29^ is designed for fuel cell-operated electric vehicles, as the topology gives the required 400 V for the wide input voltage. The voltage gain is high, but the size of the converter is larger, as three inductors are used. The topology^30^ based on a single switch, 3 diodes, 1 inductor, and 3 capacitors achieves high gain, which is less than or equal to 8.

The previously described DC-DC converters face limitations in meeting the necessary specifications for a fuel cell-powered electric car and in integrating the fuel cell stack with the electric vehicle. This article presents a dual switch high voltage gain DC-DC converter specifically designed for fuel cell electric vehicle (FCEV) applications with the aim of achieving.

- Wide low input voltage.
- High gain.
- Low input ripple current.
- Minimum components (switches, inductors, capacitors).
- High reliability by reducing voltage stress.
- Enhanced efficiency at high switching frequency (50 kHz).

All the above objectives contribute to making this converter more reliable, efficient, and suitable for fuel-cell motor vehicle applications. In FCEVs, the suggested converter is a step-up device to interface the fuel cell stack. The proposed work is divided into sections: working, analysis, control strategy, efficiency calculation and results in Sections II, III, IV, V and VI, respectively.

## Functioning of the suggested topology

### Design of the suggested work

**Fig. 2:**
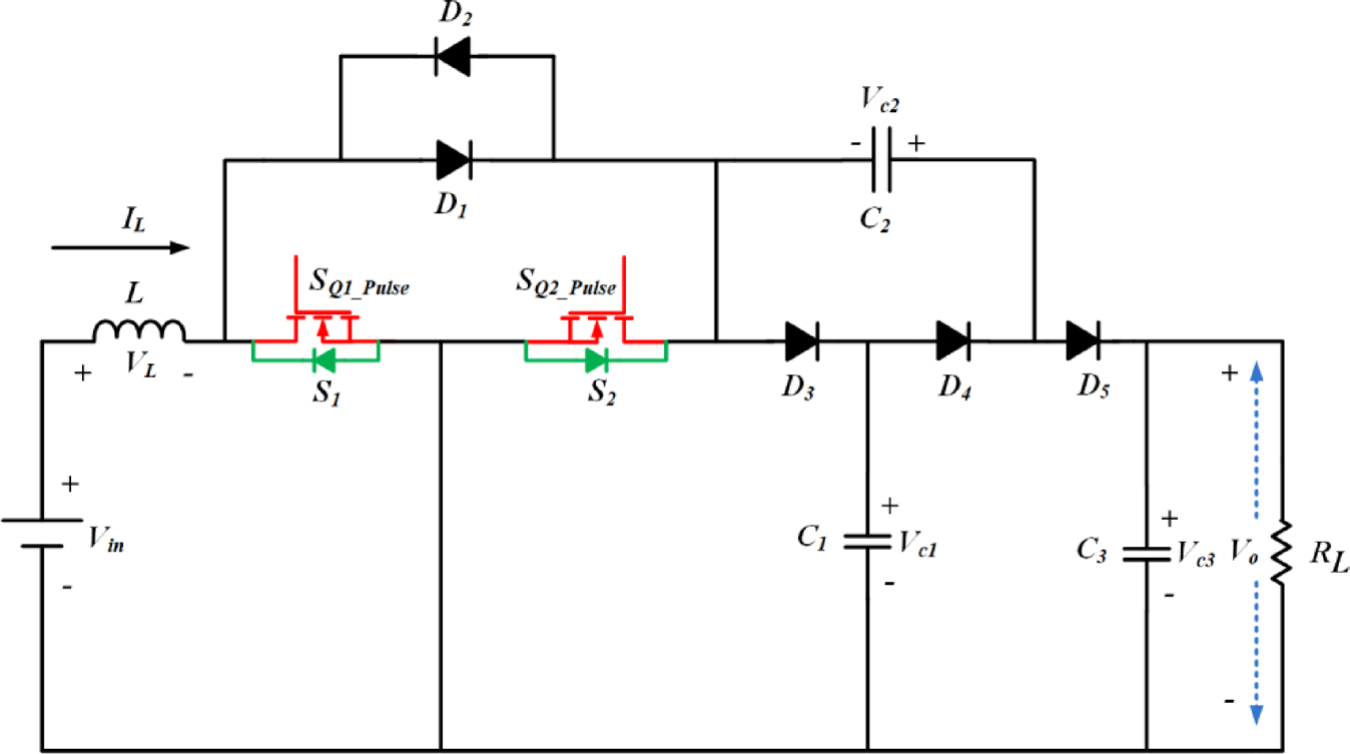
Circuit diagram of SI-SC-OSDC converter.

The proposed converter circuit diagram is shown in Fig. 2. The topology comprises three parts, namely, switched inductor (SI), switched capacitor (SC) and output with single capacitor diode (OSDC) part. On the input side, SI is connected in series to the source through the switching device *S*_1_, *S*_2_. It charges when the switches are turned on and delivers energy to the load with the help of SC and OSDC. *R*_L_ is the load resistor connected across the output capacitor *C*_3_. The SC part is constructed using two diodes *D*_3_, *D*_4_, and two capacitors *C*_1_, *C*_2_. In the SC part, *D*_3_, *D*_4_ and *C*_1_ are configured in the form of *T*-shape. In the horizontal portion of the *T*-shape, the two diodes *D*_3_, and *D*_4_ are connected in series. Capacitor *C*_1_ is connected in the vertical portion of the *T*-shape, which bifurcates the series connected diodes *D*_3_, and *D*_4_. The output diode part *D*_5_, interfaces the SC part with the load. The switched capacitor part achieves high voltage gain and limits the voltage stress on the power semiconductor components. The switched inductor part reduces the input ripple current.

**Fig. 3:**
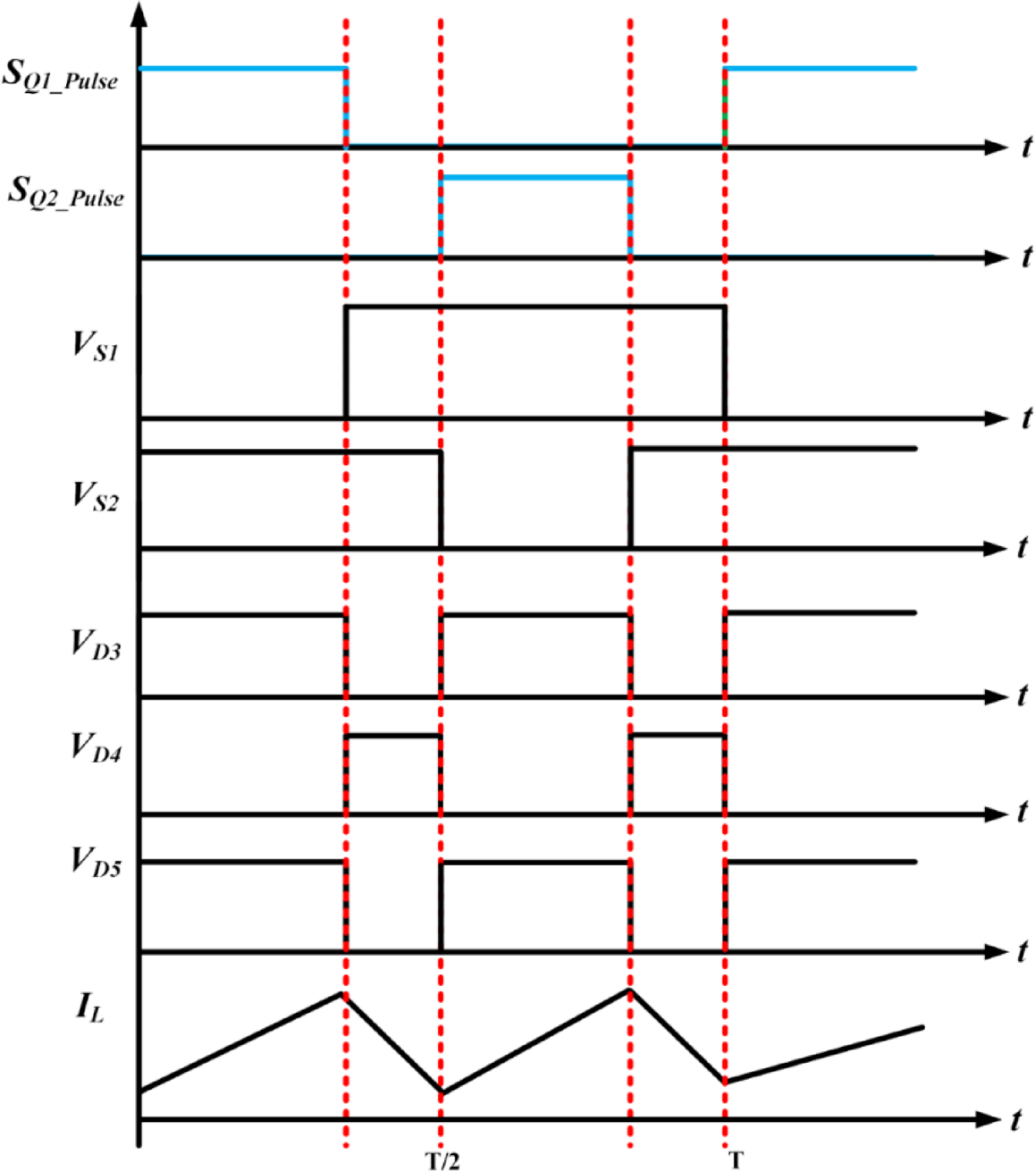
Expected waveforms of SI-SC-OSDC.

### The suggested converter’s working principle

**Fig. 4:**
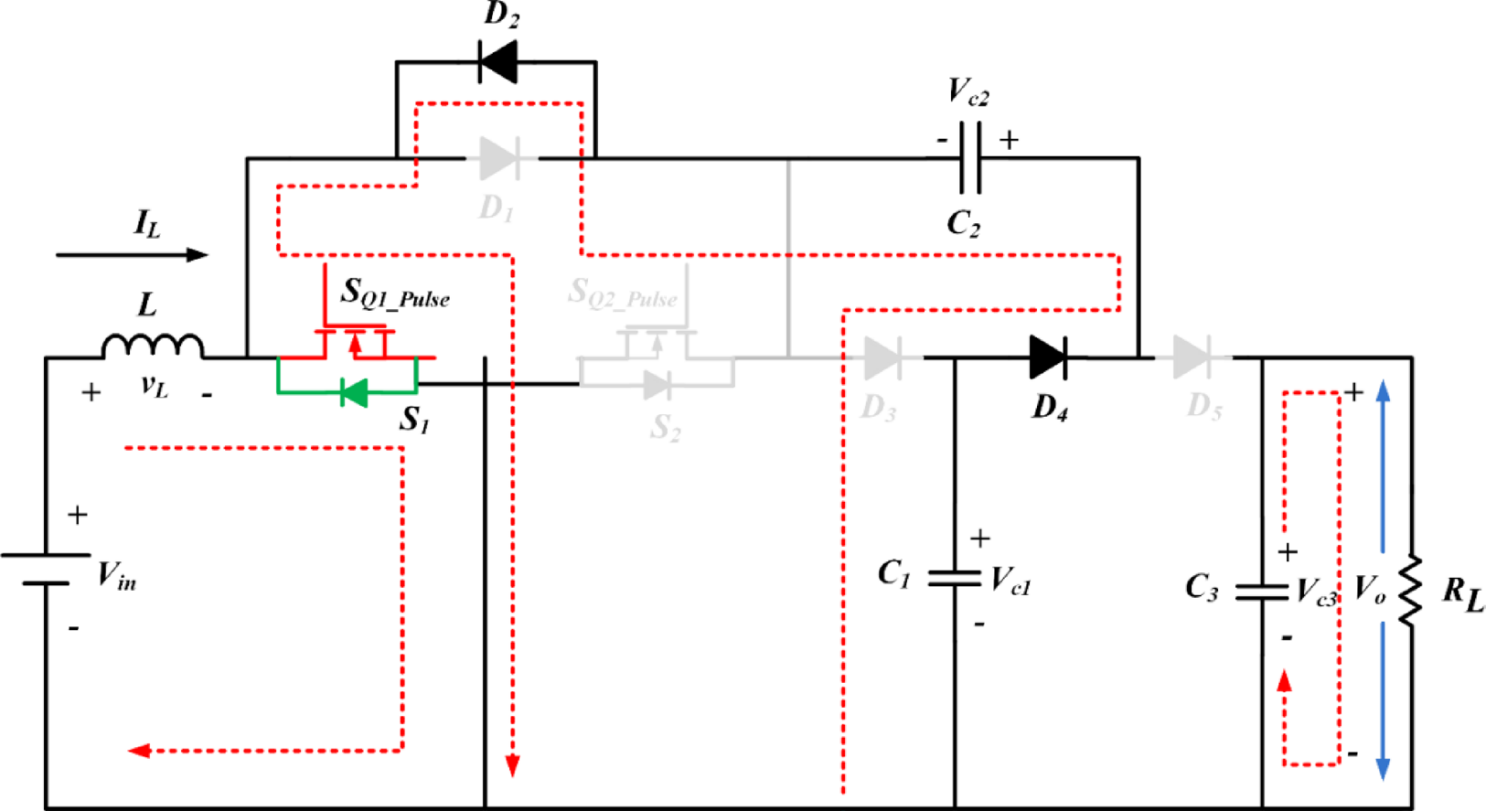
Operation of SI-SC-OSDC in mode 1.

**Fig. 5:**
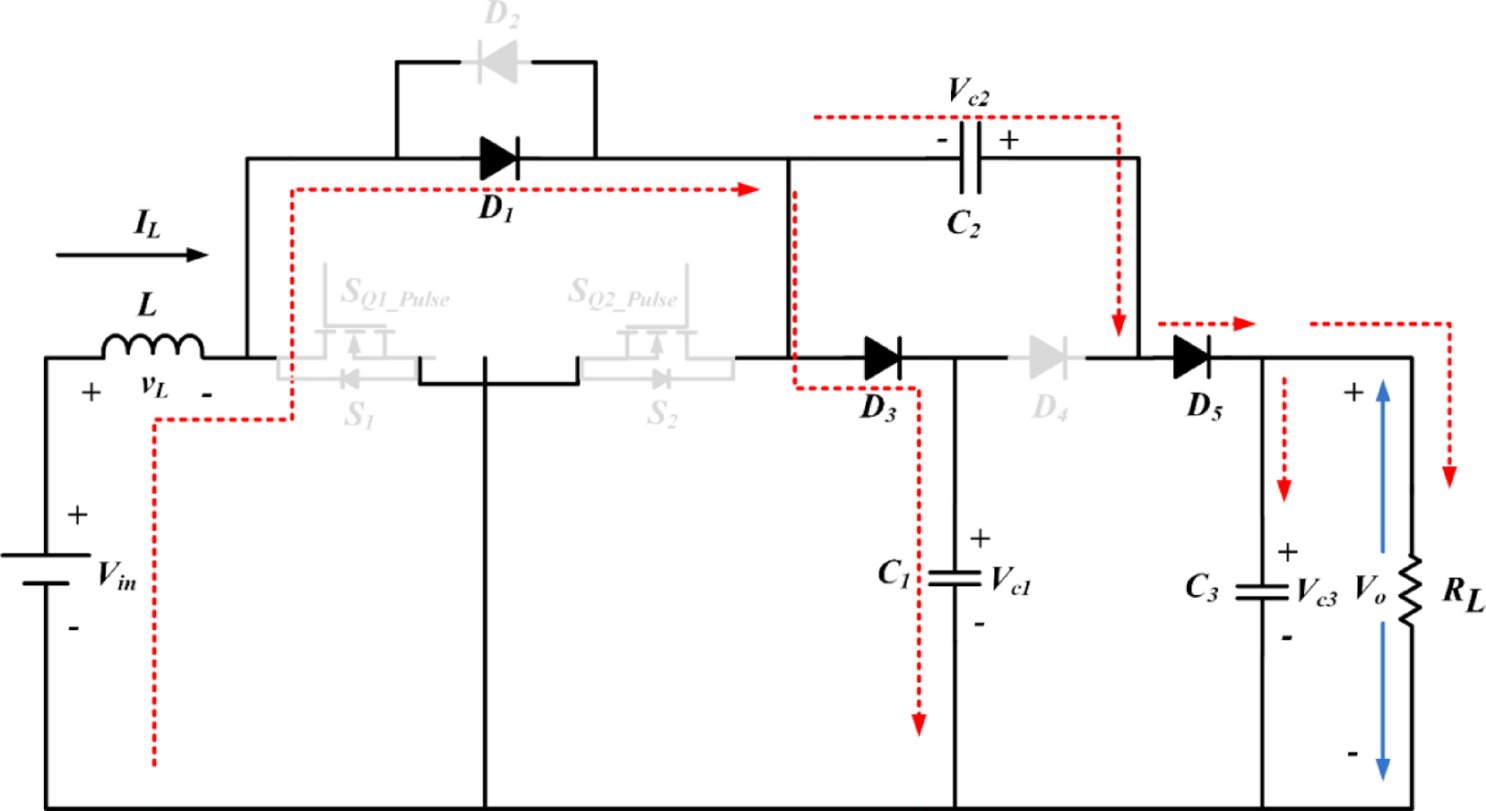
Operation of SI-SC-OSDC in mode 2 and mode 4.

The functionality of the proposed topology (SI-SC-OSDC) depicted in Fig. 3 is obtained by operating the circuit in two modes (*ON*-state-when any one of the switches is turned-*ON* / *OFF* state when both switches are turned *OFF*).

**Fig. 6:**
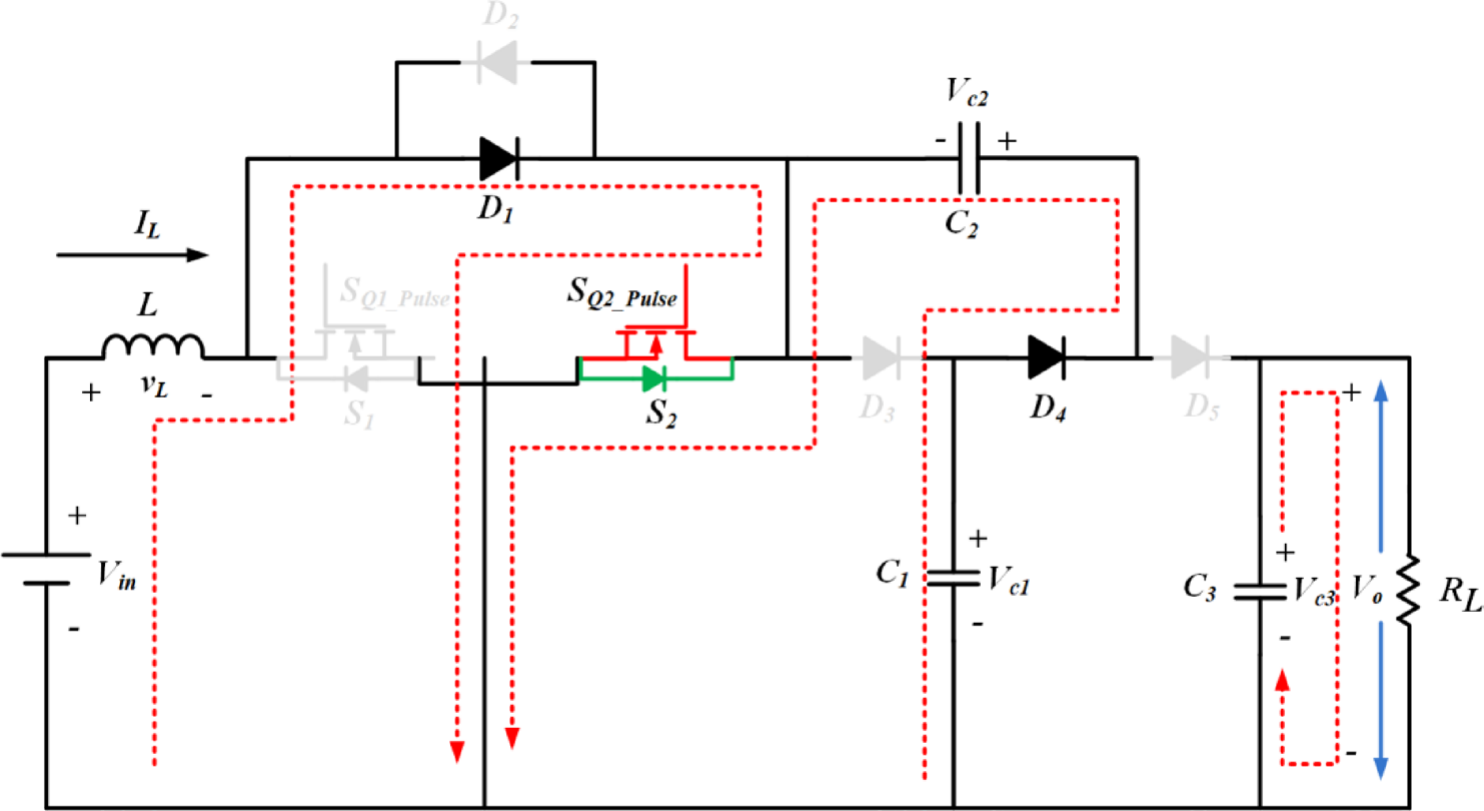
Operation of SI-SC-OSDC in mode 3.

Mode 1 & 3: Under switched *ON* condition, the third part of the circuit (OSDC) is isolated from the SI and SC parts. Hence, capacitor *C*_3_ delivers energy to the load *R*_L_. In the *ON* state of *S*_1_, *S*_2_, the inductor *L* and capacitor *C*_2_ are connected to the ground terminal. Hence, diode *D*_3_ is in *OFF* state, and the inductor charges linearly. Diode *D*_5_ is switched *OFF* due to the higher potential of capacitor *C*_3_. As one end of the terminal of capacitor *C*_2_ is connected to the ground potential, diode *D*_4_ switches *ON* due to the higher voltage magnitude (*V*_c1_) of capacitor *C*_1_. Hence, *C*_1_ charges *C*_2_ in parallel combination till the voltage of both capacitors becomes equal, and it is inferred from Figs. 4, 5 and 6.

**Fig. 7:**
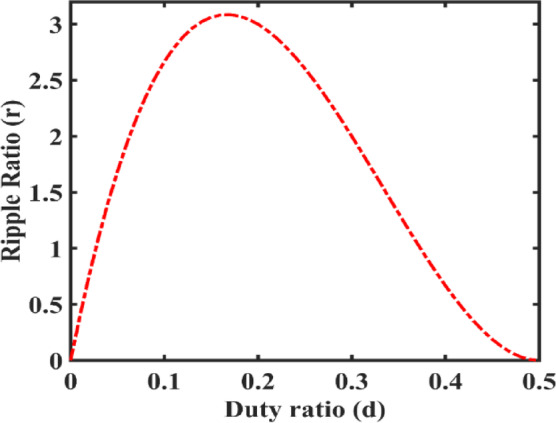
Variation of Ripple ratio (*r*) - duty ratio (*d*).

Mode 2 & 4: Under switched *OFF* state of *S*_1_, *S*_2_ as observed from Fig. 5, *D*_3_ and *D*_5_ are turned *ON*, hence *D*_4_ is turned *OFF*. Capacitor *C*_1_ of SC module is charged from SI through *D*_3_. The SI and the capacitor *C*_2_ of SC module deliver energy to the load *R*_L_ and the output capacitor *C*_3_ of OSDC module. Subsequently, the output voltage of the converter is maintained at 400 V for the wide variation of input voltage with reduced ripple current at the input side, which is inferred from Fig. 7.

Mode-1

1 $$\:\left\{\begin{array}{l}{V}_{L}={V}_{in}\\\:{V}_{c1}={V}_{c2}\\\:{V}_{o}={V}_{c3}\end{array}\right.$$

2 $$\:\left\{\begin{array}{l}{i}_{c2}={-i}_{c1}\\\:{i}_{c3}={i}_{o}\end{array}\right.$$

Mode-2

3 $$\:\left\{\begin{array}{l}{V}_{L}={V}_{in}-{V}_{c2}\\\:{V}_{c3}={V}_{c1}+{V}_{c2}\\\:{V}_{c3}={V}_{o}\end{array}\right.$$

4 $$\:\left\{\begin{array}{l}{i}_{L}={-i}_{c1}{+i}_{c2}\\\:{i}_{c3}={i}_{o}{+i}_{c3}\end{array}\right.$$

## The proposed work’s characteristics

### High voltage gain and wide input voltage

Assume that all the components are ideal and the ratings of the passive elements are sufficiently large, while also considering constant voltages across each capacitor. From the Figs. 4 and 5 output voltage *V*_o_, capacitor voltages *V*_c1_, *V*_c2_, *V*_c3_, and volt-second balance of the inductor *L* in the continuous conduction mode (CCM), the voltage gain of the proposed converter derived from Eqs. (1) and (3) is given below,

5 $$\:\left\{\begin{array}{l}{2dV}_{in}+\left({V}_{in}-{V}_{c1}\right)\times\:\left(1-2d\right)=0\\\:{V}_{o}={V}_{c3}\\\:{V}_{c1}={V}_{c2}=\frac{{V}_{c3}}{2}\end{array}\right.$$

From Eqs. (5),

6 $$\:{V}_{o}={V}_{in}\times\:\frac{2}{1-2d}$$

7 $$\:k=\frac{{V}_{o}}{{V}_{in}}=\frac{2}{1-2d}$$

8 $$\:\left\{\begin{array}{l}{V}_{o}=k\times\:{V}_{in}\:\:\:\:\:\:\left(Continuous\:Conduction\:Mode-CCM\right)\\\:{V}_{o}={V}_{in}\times\:\left(1+\sqrt{1+\frac{{d}^{2}\times\:{R}_{L}\times\:T}{2\times\:L}}\right)\:\:\left(Disontinuous\:Conduction\:Mode-DCM\right)\end{array}\right.$$

**Fig. 8:**
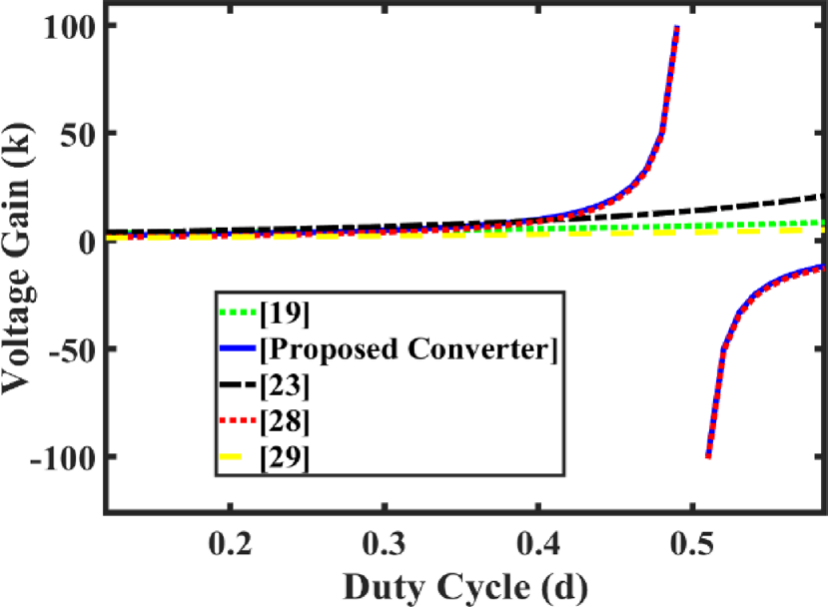
Variation of voltage gain (*k*) - duty ratio (*d*).

Where ‘*k*’ is the gain factor and ‘*d*’ is the control input (‘*d*’-duty cycle) such that ‘*d*’ is varied between 0.45 and 0.375 (0.45 > *d* > 0.375) to achieve 400 V for the variation of input voltage between 20 V and 50 V. The relation between *d*, *k*, *V*_o_, and *V*_in_ is shown in the Eq. (5) to (8), and as *d* ranges from 0.45 to 0.375, the respective voltage gain is inferred from Fig. 8.

### Ripple with low input current

By applying the ampere-second balance principle to capacitors *C*_1_, *C*_2_, and *C*_3_ in CCM, the following Eq. (9) to (12) are derived by using Eqs. (2) and (4).

9 $$\:\left\{\begin{array}{c}2dT\times\:{i}_{c1x}+\left(1-2d\right)\times\:{i}_{c1y}=0\\\:2dT\times\:{i}_{c2x}+\left(1-2d\right)\times\:{i}_{c2y}=0\\\:2dT\times\:{i}_{c3x}+\left(1-2d\right)\times\:{i}_{c3y}=0\end{array}\right.$$

*i*_c1x_ - *i*_c3x_, *i*_c1y_ - *i*_c3y_, are the currents through *C*_1_ - *C*_3_ in state 1 and state 0, respectively, and *x*, *y* refers to the state of the variable during the *ON* (1)/*OFF* (0) condition of the switch.

*I*_L_, the average value of the inductor is given in Eq. (10),

10 $$\:\begin{array}{c}{I}_{L}=\left(\frac{2}{1-2d}\right)\times\:\left(\frac{{V}_{o}}{{R}_{L}}\right)\end{array}$$

and the inductor ripple current is given by Eq. (11),

11 $$\:\begin{array}{c}{\varDelta\:i}_{L}=\left(\frac{d\times\:{V}_{in}}{L\times\:f}\right)\end{array}$$

Δ*i*_L_ = Inductor ripple current.

where *f*, is the switching frequency of the proposed converter.

Inductor ripple current ratio (‘r’) is given in Eq. (12),

r → ripple current ratio

12 $$\:r=\left(\frac{{\varDelta\:i}_{L}}{{I}_{L}}\right)=\frac{d\times\:{\left(1-2d\right)}^{2}\times\:{R}_{L}}{4\times\:L\times\:f}$$

Figure 7 depicts the variation of the current ripple ratio corresponding to the variation of the duty cycle. The ripple ratio rises to 3 at duty cycle < 0.2 and falls to zero when duty cycle is 0.5. If the circuit duty cycle operates less than 0.3, the current ripple ratio becomes more significant. At a duty ratio of 0.45, the current ripple ratio is approximately 0.25.

### Parameters design

The component inductor designed based on the Eqs. (8), (10), and (12) is represented in Eq. (13).

13 $$\:\begin{array}{c}L=\frac{d\times\:{\left(1-2\times\:d\right)}^{2}\times\:{R}_{L}}{4\times\:f\times\:r}\end{array}$$

The ripple voltage across the load is given by

14 $$\:\begin{array}{c}\varDelta\:{\mathrm{V}}_{o}=\:{\varDelta\:V}_{C3}\end{array}$$

Assume the current through all capacitors increases or decreases linearly.

Hence,

15 $$\:\left\{\begin{array}{l}{i}_{c1x}=\frac{{I}_{o}}{2d}\\\:{i}_{c1y}=-\frac{1}{\left(1-2\times\:d\right)}\times\:{I}_{o}\\\:{i}_{c2x}=-\frac{{I}_{o}}{2d}\\\:{i}_{c2y}=\frac{1}{\left(1-2\times\:d\right)}\times\:{I}_{o}\\\:{i}_{c3x}=-{I}_{o}\\\:{i}_{c3y}=\frac{2\times\:d}{\left(1-2\times\:d\right)}\times\:{I}_{o}\end{array}\right.$$

From Eqs. (14) and (15), the output voltage ripple is derived and shown in Eq. (16) and the capacitor design is presented in Eq. (17).

16 $$\:\varDelta\:{V}_{o}=\:\left(\frac{1+2\times\:d}{f\times\:C}\right)\times\:\left(\frac{{V}_{o}}{{R}_{L}}\right)$$

17 $$\:C=\:\left(\frac{1+2\times\:d}{f\times\:\varDelta\:{V}_{o}}\right)\times\:\left(\frac{{V}_{o}}{{R}_{L}}\right)$$

### Semiconductors and capacitors considering low voltage stress

The voltage stress is less than half of the output voltage across *D*_3_, *D*_4_, & *D*_5_, *S*_1_, *S*_2_, *C*_1_, and *C*_2_, but *C*_3_ is equal to output voltage because capacitor *C*_3_ is connected across the load.

According to Figs. 4, 5 and 6, either *S*_1_ or *S*_2_, *D*_4_ is in the *ON* state and *D*_3_ and *D*_5_ are in the *OFF* state, so *D*_3_, *C*_1_, and *C*_2_, *D*_5_ are connected in parallel. Hence, the voltage stress on diodes and capacitors *C*_1_ & *C*_2_ is equal. Correspondingly, other components were also obtained as follows:

18 $$\:\left\{\begin{array}{l}{V}_{D3}\:=\:{V}_{D4}={V}_{D5}=\frac{{V}_{o}}{2}\\\:{V}_{c1}\:={V}_{c2}=\frac{{V}_{o}}{2}\\\:{V}_{c3}\:={V}_{o}\end{array}\right.$$

From the above Eq. (18), except for capacitor *C*_3_ on all diodes, capacitors, and switches, voltage stress is half of the *V*_o_, whereas *V*_c3_ is equal to *V*_o_.

### Comparisons with other Step-Up solutions

In Table 1, the parameters (voltage gain, number of components, voltage and current stress of the semiconductor devices, and ripple current ratio with operating frequency) of the proposed converter are compared with different non-isolated converter topologies.

For comparison^19,23,28,29,31–34^ references are chosen based on topology construction, output voltage, components, and voltage stress. The topologies of the above-cited papers are developed using coupled inductor/capacitor switched/inductor switched/combination of switched inductor and capacitor switched. Such topologies maintained output voltage at 400 V for the variable input voltage varying between 20 V and 120 V, and voltage stress on the switches is *V*_o_/2.

In^29,32^, the presented converters achieved voltage gain in the order of 10 and 6.66 with a single switch, three inductors, five capacitors, and three diodes. In^28^ the converter is designed with four inductors, seven capacitors, and four diodes with single switch topology to obtain the voltage gain of 10. Due to the larger number of inductors in^28,29^ the core losses are higher, more EMI effect, and high inrush currents when all the inductors are charged. The converter of^23,31,33,34^ achieved voltage gain of more than 10 with reduced inductor count (two inductors). Compared to^28,29^, more diodes and switches are used by the converter of^23^. The voltage gain reported in^19^ is 16, but the input ripple current is more than 20%. The current ripple percentage reported in^29^ is low, and it is around 5%.

**Table 1:** Parameter comparison with non-isolated converters.

Ref	k	V _D_ (max)	V _S_ (max)	I _D_ (max)	I _S_ (max)	C	L	D	S	f (kHz)	*r*	d (%)
SI-SC-OSDC	$\:\frac{2}{1-2d}$ *=20*	$\:\frac{{V}_{o}}{2}$	$\:\frac{{V}_{o}}{2}$	$\:\frac{2}{1-2d}\times\:{I}_{o}\sqrt{1-d}$	$\:{I}_{o}\sqrt{d}\left(\frac{6d-1}{2d(1-2d)}\right)$	3	1	5	2	50	0.25	43.75
^19^	$\:\frac{3+d}{1-d}$ *=15.2*	$\:\frac{{2V}_{o}}{3+d}$	$\:\frac{{V}_{o}}{3+d}$	$\:{I}_{o}\sqrt{\frac{1}{1-d}}$	$\:{I}_{o}\sqrt{d}\left(\frac{2}{(1-d)}+\frac{1}{d}\right)$	4	2	4	2	50	0.25	75
^23^	$\:\frac{3+d}{{\left(1-d\right)}^{2}}$ *=10*	$\:\frac{{2V}_{o}}{3+d}$	$\:\frac{{\left(1+d\right)V}_{o}}{3+d}$	$\:\frac{2{I}_{o}}{{\left(1-d\right)}^{2}}$	$\:\frac{\left(1+3d-{d}^{2}-{d}^{3}\right)}{d{\left(1-d\right)}^{2}}\times\:{I}_{o}$	5	2	5	2	20	0.125	42
^28^	$\:\frac{1+2d}{1-2d}$ *=10*	$\:\frac{{V}_{o}}{1+2d}$	$\:\frac{{V}_{o}}{1+2d}$	$\:\left(1-d\right){I}_{o}$	$\:\frac{{4dI}_{o}}{1-2d}$	7	4	4	1	50	0.25	50
^29^	$\:\frac{1+2d}{1-d}$ *=10*	$\:\frac{{V}_{o}}{1+2d}$	$\:\frac{{V}_{o}}{1+2d}$	$\:{I}_{o}\sqrt{\frac{d}{3}}$	$\:{I}_{o}\times\:\left(\sqrt{\frac{d}{3}}\right)$	5	3	3	1	100	0.05	70
^31^	$\:\frac{4}{1-d}$ *=13.3*	$\:\frac{{V}_{o}}{2}$	$\:\frac{{V}_{o}}{2}$	$\:\frac{{I}_{in}}{2}$	$\:\frac{{I}_{in}}{2}$	4	2	4	2	100	NA	70
^32^	$\:\frac{2}{1-2d}$ *=6.6*	$\:\frac{{V}_{o}}{2}$	$\:\frac{{V}_{o}}{2}$	$\:\left(\frac{1}{1-d}\right)\times\:{I}_{o}$	$\:\left(\left(\frac{4d}{1-2d}\right)+\left(\frac{1}{d}\right)\right){I}_{o}$	4	2	4	1	20	NA	36
^33^	$\:\frac{1+nd}{{\left(1-d\right)}^{2}}$ *=10*	$\:\frac{{V}_{o}}{1+nd}$	$\:\frac{{nV}_{o}}{1+nd}$	$\:\left(\frac{1+nd}{{\left(1-d\right)}^{2}}\right)\times\:{I}_{o}$	NA	3	1	4	1	50	NA	50
^34^	$\:\frac{4}{1-d}$ *=20*	$\:\frac{{V}_{o}}{2}$	$\:\frac{{V}_{o}+{V}_{in}}{2}$	NA	NA	3	2	4	3	50	NA	50

*k*-Voltage gain, *V*_D_ (max)- Maximum diode Voltage, *V*_S_ (max)- Maximum switch-voltage, *I*_D_ (max)- Maximum Diode Current, *I*_S_ (max)- Maximum Switch Current, *C*-Capacitors, *L*-Inductors, *D*-Diodes, *S*-Switches, *f*-Switching frequency, *r*-ripple ratio, *d* − duty cycle, NA − Not Available.

The proposed work input ripple current ratio is 0.25 and achieves voltage gain of 20 with a single inductor, dual switch, three capacitors, and five diodes. Though the proposed converter produces a voltage gain of 20 compared to^19,23,28,29,31–34^ the ripple ratio and component count are less for the same voltage stress as well as for the same output voltage due to the wide variation of the input voltage. The proposed topology has reduced core losses; hence, the size and weight of the converter are also reduced, and it makes the converter more compact for the same type of power rating topologies compared to^19,23,28,29,31–34^.

## Control strategy

**Fig. 9:**
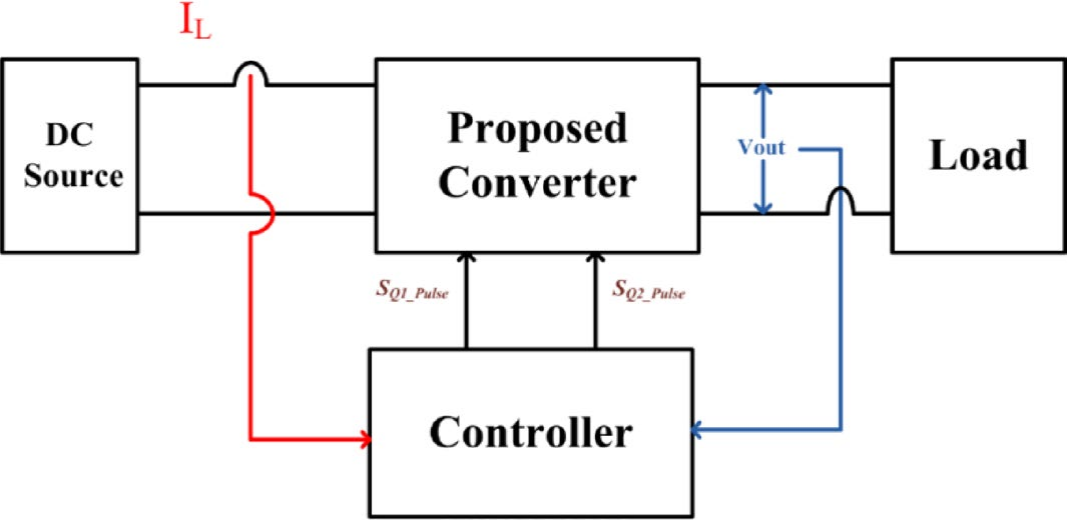
Controller representation of the SI-SC-OSDC converter.

To increase the fuel cell vehicle dynamic performance during step change and prevent shortening the life cycle of stacks due to power fluctuations, the control strategy is proposed in Fig. 9. Besides, when the electric vehicle load demands power such that the load is varied linearly, then the suggested converter supports the fuel cell to respond and supply most of the vehicle’s driving power. To stabilize the output voltage *V*_o_ at 400 V and operate the fuel cell stack in constant current output mode, two control loops, namely the outer voltage PI controller and the inner current PI controller, are used, and the design is presented in the following section.

19 $$\begin{aligned} & \left\{\left[\begin{array}{c}\dot{{i}_{L}}\\\:\dot{{v}_{c1}}\\\:\dot{{v}_{c2}}\\\:\dot{{v}_{c3}}\end{array}\right]=\left[\begin{array}{cccc}0&\:0&\:0&\:0\\\:0&\:\frac{-1}{{c}_{1}\times\:{r}_{1}}&\:\frac{-1}{{c}_{1}\times\:{r}_{1}}&\:0\\\:0&\:0&\:0&\:0\\\:0&\:0&\:0&\:\frac{-1}{{c}_{3}\times\:{R}_{L}}\end{array}\right]\left[\begin{array}{c}{i}_{L}\\\:{v}_{c1}\\\:{v}_{c2}\\\:{v}_{c3}\end{array}\right]+{v}_{in}\left[\begin{array}{c}\frac{1}{L}\\\:0\\\:0\\\:0\end{array}\right]\:\right., \\ & \quad \left[{v}_{o}\right]\:=\:\left[\begin{array}{cccc}0&\:0&\:0&\:1\end{array}\right]{\left[\begin{array}{cccc}{i}_{L}&\:{v}_{c1}&\:{v}_{c2}&\:{v}_{c3}\end{array}\right]}^{T}\end{aligned}$$

20 $$\begin{aligned} & \left\{\left[\begin{array}{c}\dot{{i}_{L}}\\\:\dot{{v}_{c1}}\\\:\dot{{v}_{c2}}\\\:\dot{{v}_{c3}}\end{array}\right]=\left[\begin{array}{cccc}0&\:0&\:\frac{-1}{L}&\:0\\\:\frac{1}{{c}_{1}}&\:\frac{-1}{{c}_{1}\times\:{r}_{1}}&\:\frac{1}{{c}_{1}\times\:{r}_{1}}&\:\frac{1}{{c}_{1}\times\:{r}_{1}}\\\:0&\:\frac{1}{{c}_{2}\times\:{r}_{1}}&\:\frac{-1}{{c}_{2}\times\:{r}_{1}}&\:\frac{1}{{c}_{2}\times\:{r}_{1}}\\\:0&\:\frac{1}{{c}_{3}\times\:{r}_{1}}&\:\frac{1}{{c}_{3}\times\:{r}_{1}}&\:-\frac{1}{{c}_{3}\times\:{R}_{L}}-\frac{1}{{c}_{3}\times\:{r}_{1}}\end{array}\right]\left[\begin{array}{c}{i}_{L}\\\:{v}_{c1}\\\:{v}_{c2}\\\:{v}_{c3}\end{array}\right]+{v}_{in}\left[\begin{array}{c}\frac{1}{L}\\\:0\\\:0\\\:0\end{array}\right]\:\right., \\ & \quad \left[{v}_{o}\right]=\left[\begin{array}{cccc}0&\:0&\:0&\:1\end{array}\right]{\left[\begin{array}{cccc}{i}_{L}&\:{v}_{c1}&\:{v}_{c2}&\:{v}_{c3}\end{array}\right]}^{T}\end{aligned}$$

21 $$\begin{aligned} & \left\{\left[\begin{array}{c}\dot{{i}_{L}}\\\:\dot{{v}_{c1}}\\\:\dot{{v}_{c2}}\\\:\dot{{v}_{c3}}\end{array}\right]=\left[\begin{array}{cccc}0&\:0&\:-\frac{1}{L}&\:0\\\:\frac{1}{{c}_{1}}&\:\frac{-2}{{c}_{1}\times\:{r}_{1}}&\:0&\:\frac{1}{{c}_{1}\times\:{r}_{1}}\\\:0&\:\frac{1}{{c}_{2}\times\:{r}_{1}}&\:\frac{-1}{{c}_{2}\times\:{r}_{1}}&\:\frac{1}{{c}_{2}\times\:{r}_{1}}\\\:0&\:\frac{1}{{c}_{3}\times\:{r}_{1}}&\:\frac{1}{{c}_{3}\times\:{r}_{1}}&\:-\frac{2}{{c}_{3}\times\:{R}_{L}}-\frac{1}{{c}_{3}\times\:{r}_{1}}\end{array}\right]\left[\begin{array}{c}{i}_{L}\\\:{v}_{c1}\\\:{v}_{c2}\\\:{v}_{c3}\end{array}\right]+{v}_{in}\left[\begin{array}{c}\frac{2}{L}\\\:0\\\:0\\\:0\end{array}\right]\right., \\ & \quad \left[{v}_{o}\right]=\left[\begin{array}{cccc}0&\:0&\:0&\:1\end{array}\right]{\left[\begin{array}{cccc}{i}_{L}&\:{v}_{c1}&\:{v}_{c2}&\:{v}_{c3}\end{array}\right]}^{T}\end{aligned}$$

### Small-signal modelling

A small signal model of the recommended converter is necessary to quantify the controller parameters. The proposed converter includes two switching states: *S*_1_*S*_2_ = [10 or 01 (*ON* state), 00 (*OFF* state)].

The converter’s operating duration in switching states of 10/01 and 00 during one cycle is *2×d×T* and *(1–2×d) ×T*, where *T* is the time period of one cycle.

The variables listed in the Eq. (22) are the steady state variables (*V*_in_, *I*_L_, *D*, *V*_c1−c3_, and *V*_o_) and small signal variables ($\:{\widehat{v}}_{in}$, $\:{\widehat{i}}_{L}$, $\:\widehat{d}$, $\:{\widehat{v}}_{c1-c3}$, and $\:{\widehat{v}}_{\mathrm{o}}$) of the corresponding quantities of the proposed topology. As a result, the state space models of the DC-DC converter in each of the three states are derived by assuming *r*_1_ as the parasitic resistance of capacitor *C*_1_ and given in Eqs. (19), (20), and (21) respectively.

Using Eqs. (19), (20), and (21), the average state space model is represented in Eq. (23). The proposed converter’s small-signal model is deduced using Eqs. (22) and (23) and expressed in Eq. (24). The output voltage to input voltage transfer function and the control input to output voltage transfer function depicted in Eqs. (25) and (26) respectively are obtained by using Eq. (24). The controller gains can be obtained by using Eq. (26).

22 $$\:\left\{\begin{array}{c}{V}_{in}={V}_{in}+{\widehat{v}}_{in}\\\:{i}_{L}={{I}_{L}+\widehat{i}}_{L}\\\:d=D+\widehat{d}\\\:{V}_{c1}={V}_{c1}+{\widehat{v}}_{c1}\\\:{V}_{c2}={V}_{c2}+{\widehat{v}}_{c2}\\\:{V}_{c3}={V}_{c3}+{\widehat{v}}_{c3}\\\:{V}_{o}={V}_{o}+{\widehat{v}}_{o}\end{array}\right.$$

23 $$\begin{aligned} & \left\{\left[\begin{array}{c}\dot{{i}_{L}}\\\:\dot{{v}_{c1}}\\\:\dot{{v}_{c2}}\\\:\dot{{v}_{c3}}\end{array}\right]=\left[\begin{array}{cccc}0&\:0&\:-\frac{1-2d}{L}&\:0\\\:\frac{1-2d}{{c}_{1}}&\:\frac{-1}{{c}_{1}\times\:{r}_{1}}&\:\frac{1-4d}{{c}_{1}\times\:{r}_{1}}&\:\frac{1-2d}{{c}_{1}\times\:{r}_{1}}\\\:0&\:\frac{1-2d}{{c}_{2}\times\:{r}_{1}}&\:\frac{2d-1}{{c}_{2}\times\:{r}_{1}}&\:\frac{1-2d}{{c}_{2}\times\:{r}_{1}}\\\:0&\:\frac{1-2d}{{c}_{3}\times\:{r}_{1}}&\:\frac{1-2d}{{c}_{3}\times\:{r}_{1}}&\:\frac{-1+2d}{{c}_{3}\times\:{r}_{1}}-\frac{1}{{c}_{3}\times\:{R}_{L}}\end{array}\right]\left[\begin{array}{c}{i}_{L}\\\:{v}_{c1}\\\:{v}_{c2}\\\:{v}_{c3}\end{array}\right]+{v}_{in}\left[\begin{array}{c}\frac{2}{L}\\\:0\\\:0\\\:0\end{array}\right]\right., \\ & \quad \left[{v}_{o}\right]=\left[\begin{array}{cccc}0&\:0&\:0&\:1\end{array}\right]{\left[\begin{array}{cccc}{i}_{L}&\:{v}_{c1}&\:{v}_{c2}&\:{v}_{c3}\end{array}\right]}^{T} \end{aligned}$$

24 $$\begin{aligned} & \left\{\left[\begin{array}{c}{\dot{\widehat{i}}}_{L}\\\:{\dot{\widehat{v}}}_{c1}\\\:{\dot{\widehat{v}}}_{c2}\\\:{\dot{\widehat{v}}}_{c3}\end{array}\right]=\left[\begin{array}{cccc}0& \:0& \:-\frac{1-2D}{L}& \:0\\\:\frac{1-2D}{{c}_{1}}& \:\frac{-1}{{c}_{1}\times\:{r}_{1}}& \:\frac{1-4D}{{c}_{1}\times\:{r}_{1}}& \:\frac{1-2D}{{c}_{1}\times\:{r}_{1}}\\\:0& \:\frac{1-2D}{{c}_{2}\times\:{r}_{1}}& \:\frac{2D-1}{{c}_{2}\times\:{r}_{1}}& \:\frac{1-2D}{{c}_{2}\times\:{r}_{1}}\\\:0& \:\frac{1-2D}{{c}_{3}\times\:{r}_{1}}& \:\frac{1-2D}{{c}_{3}\times\:{r}_{1}}& \:\frac{-1+2D}{{c}_{3}\times\:{r}_{1}}-\frac{1}{{c}_{3}\times\:{R}_{L}}\end{array}\right]\left[\begin{array}{c}{\widehat{i}}_{L}\\\:{\widehat{v}}_{c1}\\\:{\widehat{v}}_{c2}\\\:{\widehat{v}}_{c3}\end{array}\right] \right. \\ & \quad \left. +{\widehat{v}}_{in}\left[\begin{array}{c}\frac{2}{L}\\\:0\\\:0\\\:0\end{array}\right]+\left[\begin{array}{cccc}0& \:0& \:\frac{2}{L}& \:0\\\:\frac{-2}{{c}_{1}}& \:0& \:\frac{-4}{{c}_{1}\times\:{r}_{1}}& \:\frac{-2}{{c}_{1}\times\:{r}_{1}}\\\:0& \:\frac{-2}{{c}_{2}\times\:{r}_{1}}& \:\frac{2}{{c}_{2}\times\:{r}_{1}}& \:\frac{-2}{{c}_{2}\times\:{r}_{1}}\\\:0& \:\frac{-2}{{c}_{3}\times\:{r}_{1}}& \:\frac{-2}{{c}_{3}\times\:{r}_{1}}& \:\frac{2}{{c}_{3}\times\:{r}_{1}}\end{array}\right]\left[\begin{array}{c}{I}_{L}\\\:{V}_{c1}\\\:{V}_{c2}\\\:{V}_{c3}\end{array}\right]\widehat{d}\: \right. \\ & \quad \left. \left[{\widehat{v}}_{o}\right]=\left[\begin{array}{cccc}0& \:0& \:0& \:1\end{array}\right]{\left[\begin{array}{cccc}{\widehat{i}}_{L}& \:{\widehat{v}}_{c1}& \:{\widehat{v}}_{c2}& \:{\widehat{v}}_{c3}\end{array}\right]}^{T}\right. \end{aligned}$$

25 $$\:{G}_{{v}_{o}{v}_{in}}\left(s\right)=\frac{{\widehat{v}}_{0}\left(s\right)}{{\widehat{v}}_{in}\left(s\right)}{|}_{\widehat{d}\left(s\right)=0}=\frac{1.102\times\:{10}^{12}\times\:s+6.678\times\:{10}^{16}}{{s}^{4}+3.637\times\:{10}^{5}\times\:{s}^{3}+2.48\times\:{10}^{10}\times\:{s}^{2}+3.9\times\:{10}^{14}\times\:s+3.34\times\:{10}^{15}}$$

26 $$\:{G}_{{v}_{o}d}\left(s\right)=\frac{{\widehat{v}}_{0}\left(s\right)}{\widehat{d}\left(s\right)}{|}_{{\widehat{v}}_{in}\left(s\right)=0}=\frac{-7.359\times\:{10}^{12}\times\:{s}^{2}-4.458\times\:{10}^{17}\times\:s+2.672\times\:{10}^{19}}{{s}^{4}+3.638\times\:{10}^{05}\times\:{s}^{3}+2.481\times\:{10}^{10}\times\:{s}^{2}+3.901\times\:{10}^{14}\times\:s+3.341\times\:{10}^{15}}$$

**Table 2:** SI-SC-OSDC parameters.

S. no	Parameters	Values
1	Input Voltage (*V*_s_ = *V*_in_)	20–50 V
2	Output Voltage (*V*_o_)	400 V
3	Inductor (*L*)	50 µH
4	Capacitor (*C*_1_, *C*_2_, *C*_3_)	110 µF
5	Switching Frequency (*f*)	50 kHz
6	Load (*R*_L_ = 400 Ω)	1 A, 400 W
7	Power switches	IXFP80N25 × 3
8	Power Diode	MBR40250G

## Efficiency calculations

The converter’s efficiency, as indicated by Eq. (27), together with the other power loss components, is presented below by using equations (28) to (34).

27 $$\:\%\:\eta\:\:=\frac{{P}_{in}-{P}_{QR}-{P}_{SL}-{P}_{D}-{P}_{Cu}-{P}_{C}}{{P}_{in}}\times\:100\:$$

The rms values of the currents of the controlled and uncontrolled power switches are shown in Eqs. (28),

28 $$\:\left\{\begin{array}{l}{{I}_{Q1\left(rms\right)}=\:{I}_{Q2\left(rms\right)}=\:I}_{o}\times\:\sqrt{d}\times\:\left(\frac{6d-1}{2d(1-2d)}\right)\\\:{I}_{D1\left(rms\right)}={\frac{2}{1-2d}\times\:I}_{o}\times\:\sqrt{1-d}\\\:\:{I}_{D2\left(rms\right)}={I}_{D4\left(rms\right)}=\frac{1}{2d}\times\:\sqrt{d}\:\\\:{I}_{D3\left(rms\right)}=\:\frac{{I}_{o}}{1-2d}\times\:\sqrt{1-d}\\\:{I}_{D5\left(rms\right)}=\frac{1}{2d}\times\:\sqrt{1-d}\end{array}\right.$$

The conduction loss (*P*_QR_) and switching losses (*P*_SL_ = turn on *P*_on_ and turn off losses *P*_off_) contribute to the total amount of power loss of the controlled switching devices and are expressed in the following Eqs. (29) and (30) respectively.

Here, *I*_Q1−DM_ represent the currents flowing through switch *Q* when it is turned on and *V*_DM_ refers to the voltage when the power switch is turned off, and power switch on-state resistance is *r*_Q_, diode forward resistance is *r*_D_.

29 $$\:\left\{\begin{array}{l}{P}_{QR1}=\:{{\left({I}_{Q1\left(rms\right)}\right)}^{2}\times\:{r}_{Q}}_{\:}\\\:{P}_{on}=\:\frac{1}{2}\times\:{V}_{DM}\times\:{I}_{QDM}\times\:f\times\:\left({t}_{2}+{t}_{3}\right)\\\:\:{P}_{off}=\frac{1}{2}\times\:{V}_{DM}\times\:{I}_{QDM}\times\:f\times\:\left({t}_{4}+{t}_{5}\right)\:\end{array}\right.$$

30 $$\:\left\{\begin{array}{l}{P}_{QR2}=\:{{\left({I}_{Q2\left(rms\right)}\right)}^{2}\times\:{r}_{Q}\:}_{\:}\\\:{P}_{QR}=\:{P}_{QR1}+{P}_{QR2}=\:\left({\left({I}_{Q1\left(rms\right)}\right)}^{2}+\:{\left({I}_{Q2\left(rms\right)}\right)}^{2}\right)\times\:{r}_{Q}\\\:\:{P}_{SL}={P}_{ON}+{P}_{off}={V}_{DM}\times\:{I}_{DM}\times\:\left({t}_{2}\:+\:{t}_{3}+{t}_{4}+{t}_{5}\right)\times\:f\:\end{array}\right.$$

The three types of power losses that may occur in a diode are the loss due to diode forward voltage *P*_DF_, conduction loss *P*_DR_, and reverse recovery loss. The remaining loss namely, the diode switching losses *P*_D_ are disregarded for the calculation and by using Eq. (32), the diode loss is projected in Eq. (33).

31 $$\:\left\{\begin{array}{c}{I}_{D1-Avg}={V}_{DF1}\times\:\frac{{V}_{o}}{{R}_{L}}\\\:{I}_{D2-Avg}={V}_{DF2}\times\:\frac{{V}_{o}}{{R}_{L}}\\\:{I}_{D3-Avg}={V}_{DF3}\times\:\frac{{V}_{o}}{{R}_{L}}\\\:{I}_{D4-Avg}={V}_{DF4}\times\:\frac{{V}_{o}}{{R}_{L}}\\\:{I}_{D5-Avg}={V}_{DF5}\times\:\frac{{V}_{o}}{{R}_{L}}\end{array}\right.$$

32 $$\:\left\{\begin{array}{c}{P}_{DF1}={V}_{DF1}\times\:\frac{{V}_{o}}{{R}_{L}}\\\:{P}_{DF2}={V}_{DF2}\times\:\frac{{V}_{o}}{{R}_{L}}\\\:{P}_{DF3}={V}_{DF3}\times\:\frac{{V}_{o}}{{R}_{L}}\\\:{P}_{DF4}={V}_{DF4}\times\:\frac{{V}_{o}}{{R}_{L}}\\\:{P}_{DF5}={V}_{DF5}\times\:\frac{{V}_{o}}{{R}_{L}}\end{array}\right.$$

Here, the average current flowing through diodes *D*_1_ − *D*_5_ during one cycle is denoted by *I*_D1,ave_ -I_D3,ave_ and are shown in Eq. (31). The diode’s current has a negligible effect on the dynamic internal resistance *r*_D_. Due to this, the conduction resistance *P*_DR_ loss of the diode is insignificant compared to the loss of the diode forward voltage *P*_DF_.

33 $$\:\left\{\begin{array}{l}{P}_{DF}={P}_{DF1}+\:{P}_{DF2}\:+{P}_{DF3}+{P}_{DF4}+{P}_{DF5}\\\:{P}_{DR}=\:{\left({\left({I}_{D1\left(rms\right)}\right)}^{2}+{\left({I}_{D2Q\left(rms\right)}\right)}^{2}+{\left({I}_{D3\left(rms\right)}\right)}^{2}+{\left({I}_{D4\left(rms\right)}\right)}^{2}+{\left({I}_{D5\left(rms\right)}\right)}^{2}\right)\times\:{r}_{D}}_{\:}\end{array}\right.$$

Finally, the losses of inductor and capacitor (*P*_Cu_ - Copper losses of inductor and *P*_C_ - losses due to series resistance of capacitor) are calculated by using Eqs. (34),

34 $$\:\left\{\begin{array}{l}{P}_{{C}_{u}}=\:{{\left({I}_{L}\right)}^{2}\times\:{r}_{L}\:}_{\:}\:\\\:{P}_{c}=\:{\left({\left({I}_{RC1\left(rms\right)}\right)}^{2}+{\left({I}_{RC2\left(rms\right)}\right)}^{2}+{\left({I}_{RC3\left(rms\right)}\right)}^{2}\right)\times\:{r}_{c}\:}_{\:}\end{array}\right.$$

## Results of proposed work

The results of SI-SC-OSDC with respect to Fig. 2/Fig. 9 and as per the parameters of Table 2 are depicted in Figs. 10, 11, 12, 13, 14, 15, 16, 17 and 18. Hardware setup of proposed SI-SC-OSDC is shown in Fig. 10.

The steady-state load voltage and the inductor current are shown in Figs. 11 and 12. With the reference voltage set to 400 V and with 20 V at the input source side, 400 V is obtained at the load, as observed in Fig. 11. The inductor current depicted along with the switching pulse indicates that the inductor charges linearly and discharges linearly with the duty ratio of 0.45, and the change in *i*_L_ is 5.4 A for the average inductor current of 21.8 A.

**Fig. 10:**
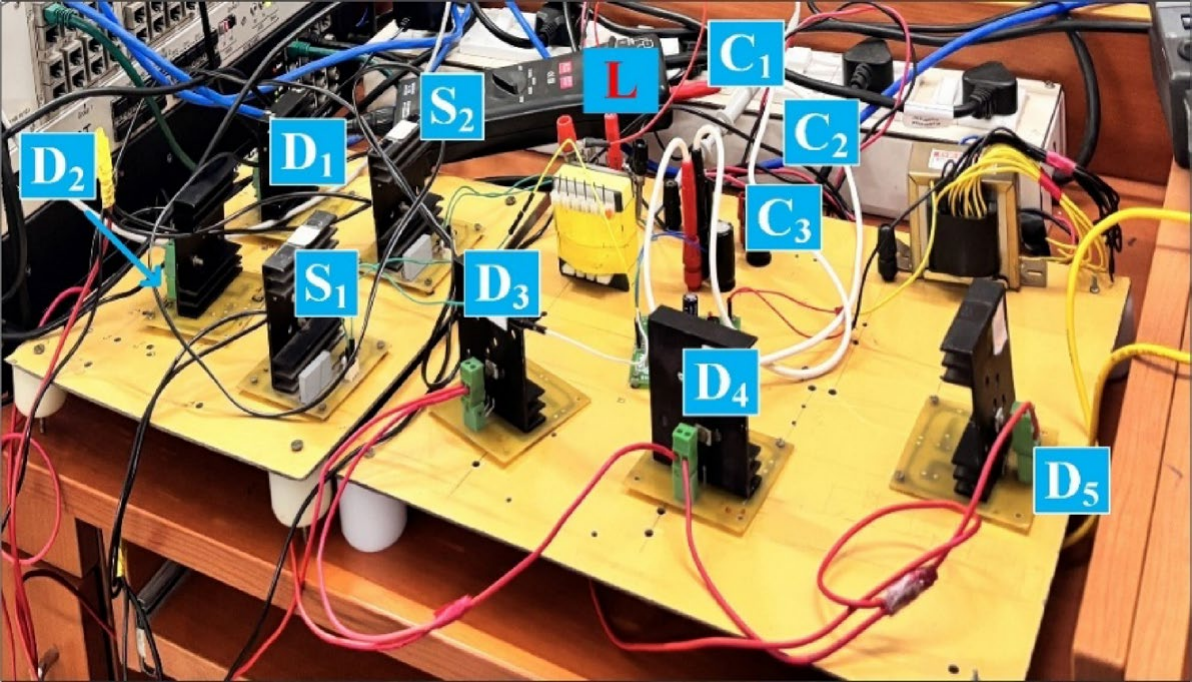
Hardware setup of the proposed topology SI-SC-OSDC.

**Fig. 11:**
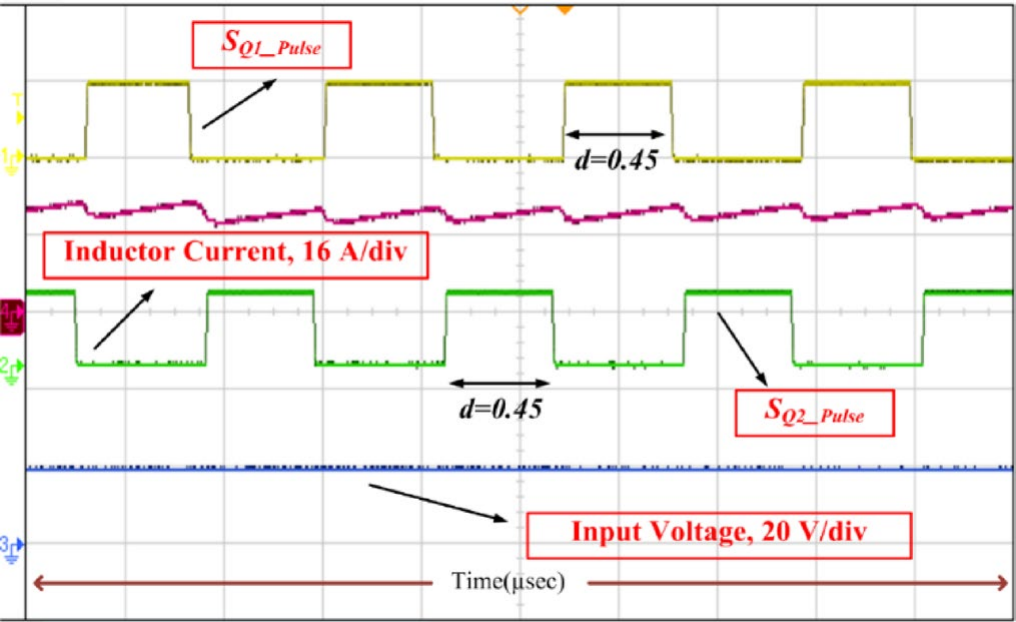
Inductor current *i*_L_ along with switching pulse and *V*_in_ when *d* = 0.45.

**Fig. 12:**
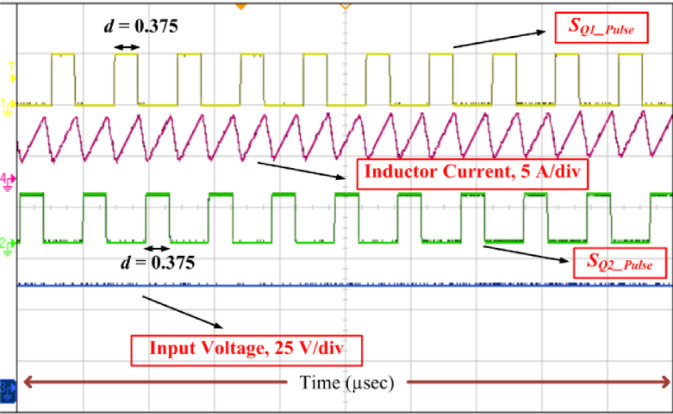
Inductor current *i*_L_ along with switching pulse and *V*_in_ when *d* = 0.375.

The feasibility of the proposed topology (SI-SC-OSDC) is tested by redoing the above procedure for another operating point such that the control input (duty ratio) is 0.375, and it can be observed in Fig. 12. Here, the gain factor is 8, whereas the gain factor for the test condition depicted in Fig. 11 is 20. To meet the requirements of a high voltage gain converter, voltage stress across the capacitors, controlled, and uncontrolled semiconductor devices will increase. But the proposed converter reduces that voltage stress to 50% of the *V*_o_. Moreover, the inductor and capacitors of the converter used for constructing the topology are reduced to nearly half the size of the converters mentioned in the Table 2.

**Fig. 13:**
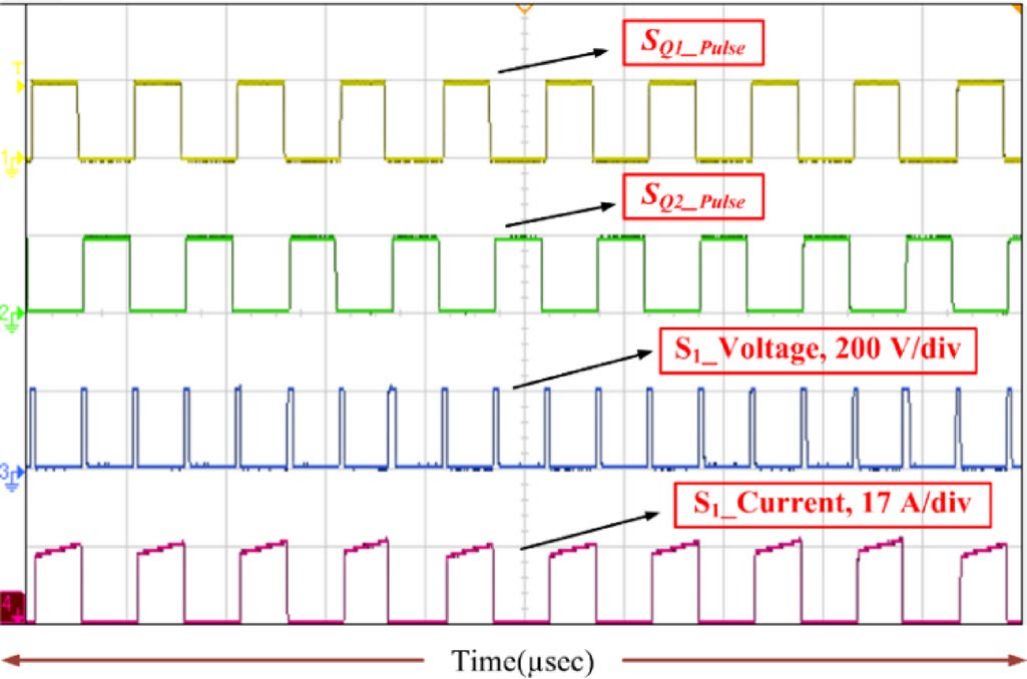
Voltage stress (*V*_S1_) and current stress (*i*_S1_) on switch with switching pulse.

**Fig. 14:**
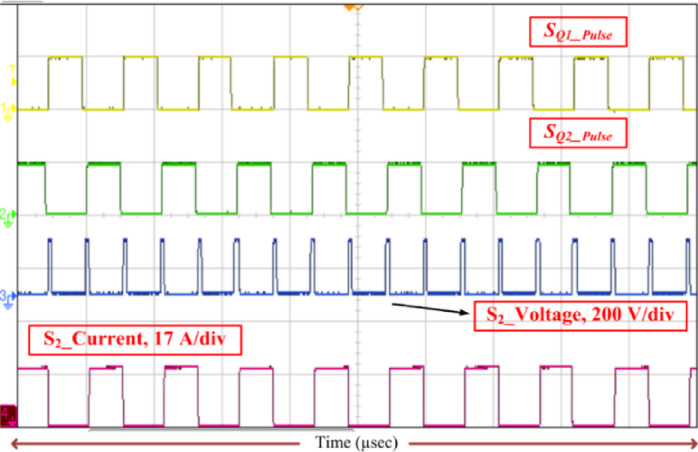
Voltage stress (*V*_S2_) and current stress (*i*_S2_) on switch with switching pulse.

**Fig. 15:**
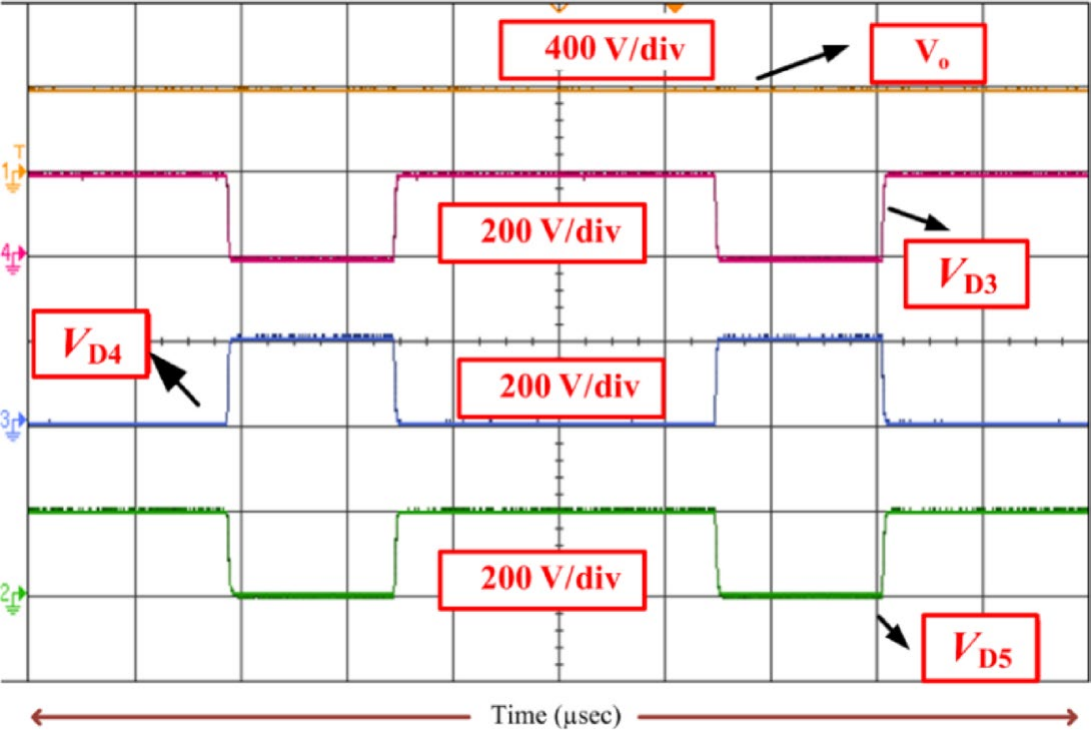
Voltage across diodes (*V*_D3_, *V*_D4_, and *V*_D5_) when *k = 20*.

**Fig. 16:**
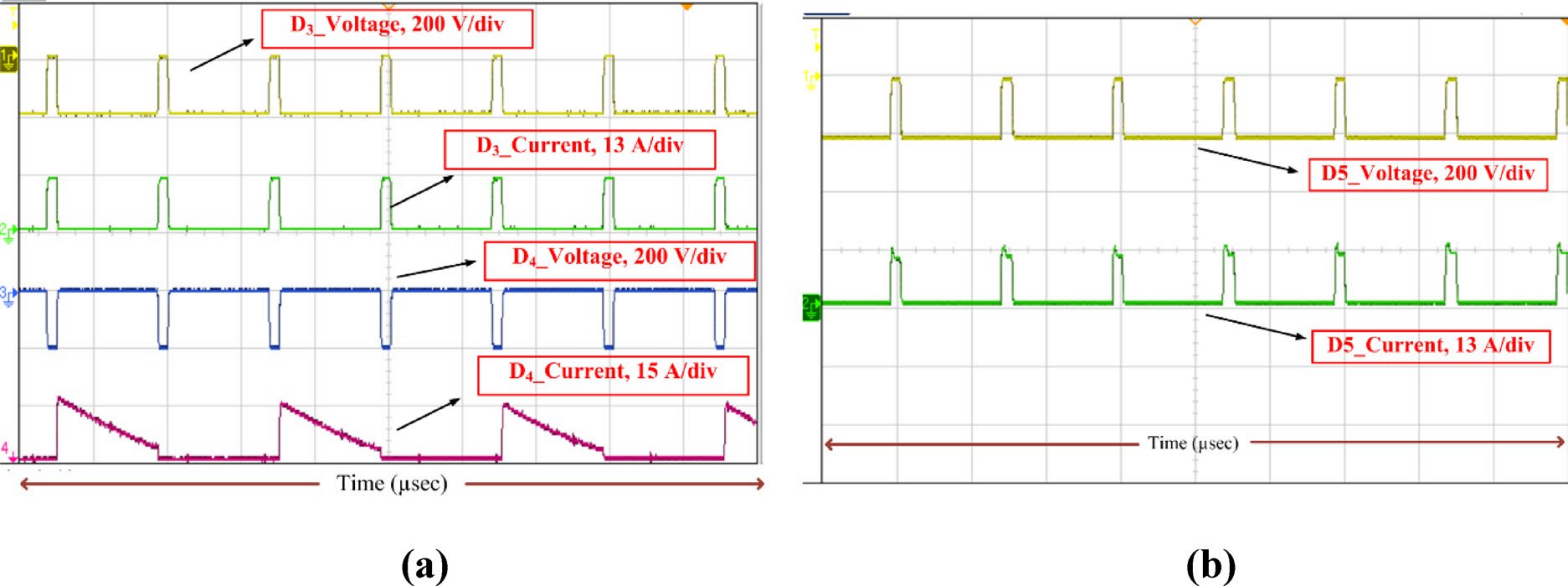
Voltage-current stress of the diodes (*D*_3_, *D*_4_, *D*_5_). (**a**) Voltage (*V*_D3_, *V*_D4_) and current stress (*i*_D3_, *i*_D4_) -Diode of SC module of SI-SC-OSDC, (**b**) Voltage stress (*V*_D5_) and current stress (*i*_D5_) -Diode (*D*_5_), of SC-OSDC module of SI-SC-OSDC.

**Fig. 17:**
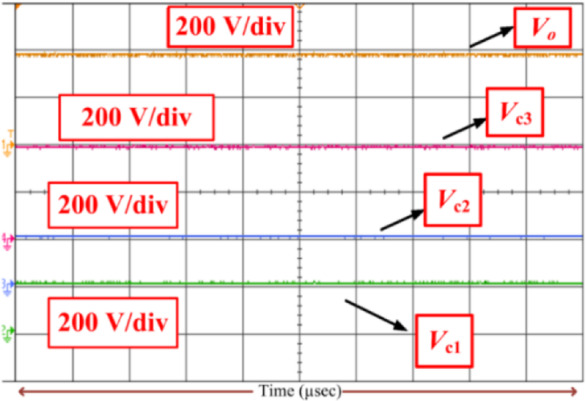
Voltage across capacitors (*V*_c1_, *V*_c2_, and *V*_c3_) when *k = 20*.

Consequently, the voltage stress across the controlled and uncontrolled semiconductor devices and capacitors is projected in Figs. 13, 14, 15, 16 and 17. From Fig. 13, with respect to the switching pulse, the voltage stress of the controlled semiconductor device *S*_1_ is 200 V, and the current through the device is 15 A when the gain of the converter is 20. Similarly, the voltage stress of the controlled devices *S*_2_ is shown in Fig. 14. Figures 15 and 16 portray the voltage across the diodes *D*_3_, *D*_4_, and *D*_5_ and current through the diodes, respectively. The voltage stress across the capacitors *C*_1_, *C*_2_, and *C*_3_ is equal to 50% of the *V*_o_ is inferred from Fig. 17.

It is evident from Figs. 13, 14, 15, 16 and 17 that the voltage stress across the controlled and uncontrolled semiconductor devices and capacitors is equal to 50% of the *V*_o_ except on *C*_3_ because *C*_3_ is connected in parallel to the load and the currents through the diodes *D*_3_, *D*_4_, and *D*_5_ presented in Fig. 16a and b are less than 15 amps.

**Fig. 18:**
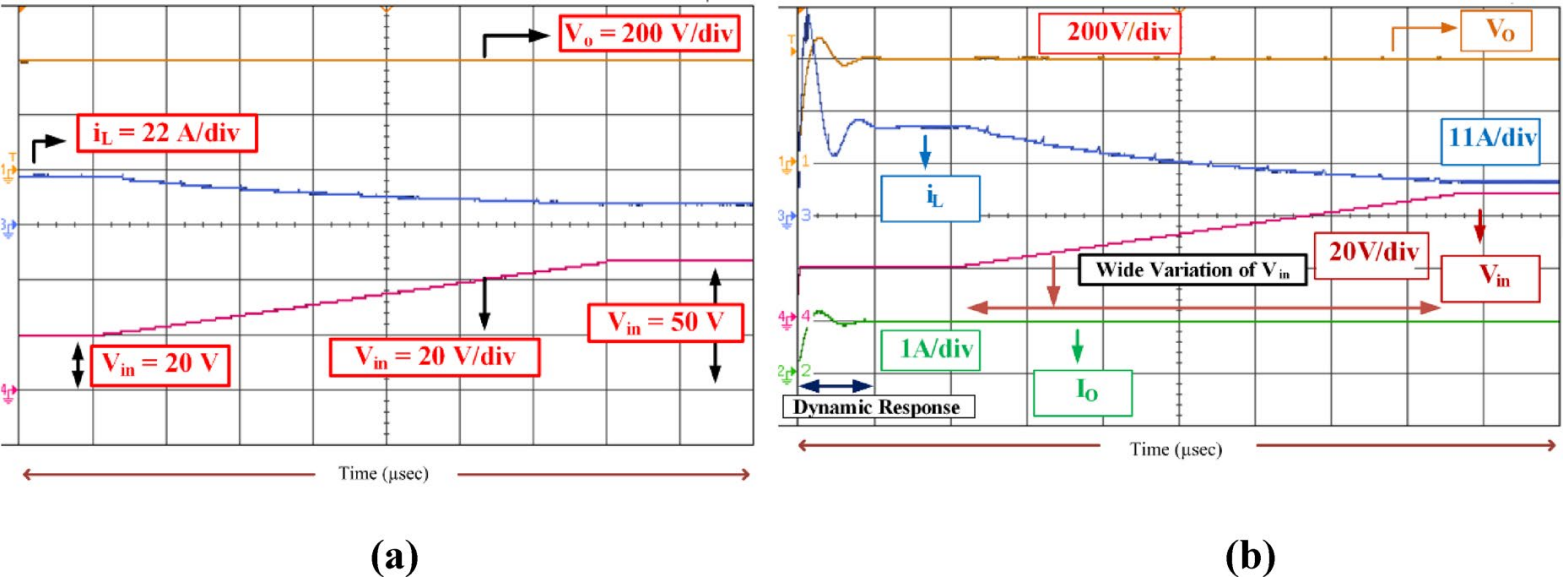
Response of SI-SC-OSDC. (**a**) Converter dynamic response-at starting and wide variation of input voltage (*V*_in_, *i*_L_, *V*_o_, and *I*_o_), (**b**) output voltage *V*_o_ and inductor current *i*_L_ response for the wide variation of input voltage *V*_in_.

The above results validate the feasibility of the proposed topology under two operating points. To further enhance the performance of the converter under wide variation of the input voltage of the fuel cell, a soft testing method is carried out. The soft testing result is shown in Fig. 18. Figure 18a portrays the dynamics of the converter besides the wide variation of input voltage. From the responses of *i*_L_, *I*_o_, and *V*_o_ with respect to the input voltage of 20 V, the load voltage is maintained at 400 V, hence it proves the effectiveness of the controller. To further substantiate the wide variation of the input voltage, the source voltage is varied from 20 V to 50 V at a rate of 8 s. Throughout the variation of the input voltage between 20 V and 50 V, the load voltage is maintained constant at 400 V, as can be inferred from Fig. 18a and b.

**Fig. 19:**
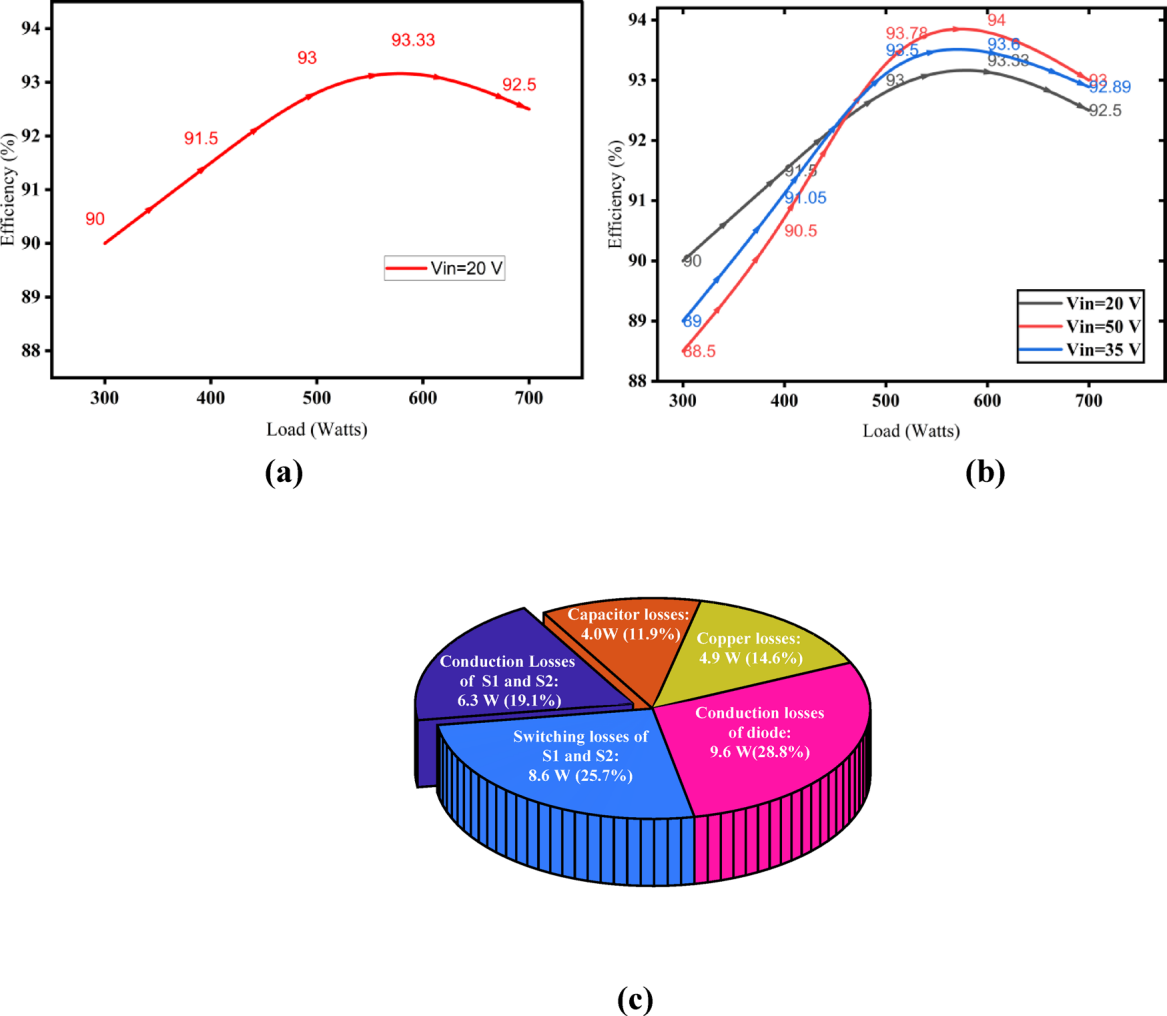
Efficiency and loss distribution of SI-SC-OSDC. (**a**) Converter efficiency under various load conditions when the voltage gain is set to 20. (**b**) Converter efficiency under various load conditions and at different voltage gain; *k* = *8*,* 11.42*, and *20*. (**c**) Calculated loss distribution.

The efficiency of the proposed converter was assessed under a range of load conditions for different input voltages, as illustrated in Fig. 19. Setting the input voltage to 20 with a voltage gain of 20, as depicted in Fig. 19a, demonstrates the efficiency across different load conditions. Figure 19b demonstrates the converter’s efficiency across multiple load conditions for three distinct input voltages. The proposed converter achieves 93.33% efficiency when the load is 600 W and 91.5% efficiency when the load is 400 W, as shown in Fig. 19a. The losses corresponding to 400 W is shown in Fig. 19c. Figure 19b illustrates the efficiency of the converter under various input voltages and load conditions. The proposed converter achieves a high efficiency of 93.33% at 600 W. It is evident that from Fig. 19b, the efficiency of the proposed topology is maximum when the input voltage is 50 V.

## Conclusion

This work proposes a revolutionary non-isolated boost converter with minimal components and high voltage gain for broad input voltage fluctuation. It reduces reverse recovery losses by minimizing the number of diodes and imposing a voltage stress of less than half the output voltage on the diodes. It has a lower ripple effect and provides common ground. This converter was tested with a broad range of input voltages ranging from 20 to 50 V to obtain a high voltage gain (20 to 8) while maintaining a load voltage of 400 V with a maximum efficiency of 93.33%. This converter is suited for use in electric vehicles powered by fuel cells.

## Data Availability

The datasets used and/or analyzed during the current study are available in the manuscript.
